# Imaging short- and long-term training success in chronic aphasia

**DOI:** 10.1186/1471-2202-10-118

**Published:** 2009-09-22

**Authors:** Ricarda Menke, Marcus Meinzer, Harald Kugel, Michael Deppe, Annette Baumgärtner, Hagen Schiffbauer, Marion Thomas, Kira Kramer, Hubertus Lohmann, Agnes Flöel, Stefan Knecht, Caterina Breitenstein

**Affiliations:** 1 Department of Neurology, University of Münster, Münster, Germany; 2 Department of Clinical Neurology, Oxford University, Oxford, UK; 3 Department of Clinical & Health Psychology, University of Florida, Gainesville, USA; 4 Department of Clinical Radiology, University of Münster, Münster, Germany; 5 Department of Health Sciences, Hochschule Fresenius, University of Applied Sciences, Hamburg, Germany; 6 Neurocenter, Schön Klinik, Hamburg, Germany

## Abstract

**Background:**

To date, functional imaging studies of treatment-induced recovery from chronic aphasia only assessed short-term treatment effects after intensive language training. In the present study, we show with functional magnetic resonance imaging (fMRI), that different brain regions may be involved in immediate versus long-term success of intensive language training in chronic post-stroke aphasia patients.

**Results:**

Eight patients were trained daily for three hours over a period of two weeks in naming of concrete objects. Prior to, immediately after, and eight months after training, patients overtly named trained and untrained objects during event-related fMRI. On average the patients improved from zero (at baseline) to 64.4% correct naming responses immediately after training, and treatment success remained highly stable at follow-up. Regression analyses showed that the degree of short-term treatment success was predicted by increased activity (compared to the pretraining scan) bilaterally in the hippocampal formation, the right precuneus and cingulate gyrus, and bilaterally in the fusiform gyri. A different picture emerged for long-term training success, which was best predicted by activity increases in the right-sided Wernicke's homologue and to a lesser degree in perilesional temporal areas.

**Conclusion:**

The results show for the first time that treatment-induced language recovery in the chronic stage after stroke is a dynamic process. Initially, brain regions involved in memory encoding, attention, and multimodal integration mediated treatment success. In contrast, long-term treatment success was predicted mainly by activity increases in the so-called 'classical' language regions. The results suggest that besides perilesional and homologue language-associated regions, functional integrity of domain-unspecific memory structures may be a prerequisite for successful (intensive) language interventions.

## Background

Chronic aphasic symptoms affect approximately 18 percent of stroke patients [1]. Language abilities can be significantly improved in the chronic stage after a stroke when training is sufficiently intensive with more than five hours of training per week [2], even when total language training lasts for merely two weeks [3-6]. Several recent functional brain imaging studies investigated the neural correlates of *spontaneous *language recovery in aphasia patients, showing the importance of perilesional areas for (covert) language production [7]. Additionally, a dominant right-sided activity pattern was observed for the least recovered patients in the chronic stage after stroke and has been interpreted in some studies as a less effective compensatory strategy [8]. Similarly, in a longitudinal study that followed up patients from the acute to the chronic stage, an early upregulation of right-hemisphere activity after stroke was followed by reactivation of left frontal areas in the chronic stage in well recovered patients [9]. However, right-sided activity patterns can also be functionally relevant, at least for the recovery of language comprehension [10].

The few available intensive *intervention *case studies using functional imaging (for a recent review see [11]) yielded highly heterogeneous recovery patterns across individual patients [12-17], making it difficult to draw general conclusions about brain areas required for the success of intensive interventions. Only three very recent studies assessed the neural substrates of intensive language training success at the group level. Raboyeau et al. [18] determined changes of brain activity measured with positron-emission-tomography (PET) in ten aphasia patients prior to and after four weeks of (lexical) training. Better performance after training was correlated with activity increases in right-sided language (insula) and frontal attention areas. A training-induced modulation of right frontal areas was also observed in the recent functional magnetic resonance (fMRI) study by Richter and colleagues [5]. On the other hand, Meinzer et al. [19] found increased fMRI activity after training selectively in individually determined perilesional brain areas in eleven chronic aphasia patients, and only activity increases in these perilesional (predominantly left frontal) areas correlated with intensive therapy success.

One caveat of intensive intervention neuroimaging studies to date is that only short-term treatment effects were assessed. Like other types of declarative memory, the long-term consolidation of language information may require different brain areas than the ones participating in initial learning [20-22]. Evidence for dynamic shifts within neural networks over time stems from recent language learning studies with healthy subjects showing that brain structures involved in general learning, such as the hippocampus, mediate the *initial *learning of lexical, semantic, and syntactic knowledge [23-27]. During later stages of memory consolidation, secondary associative cortices may be functionally required [22,25].

Here, we aimed to differentiate for the first time brain regions mediating successful short-term versus successful long-term therapy success in a group of chronic aphasia patients. We hypothesized that a network of memory and attention-related brain regions - similar to the one observed in healthy subjects during word learning [e.g. [25]] and in aphasia patients immediately after intensive language therapy [5,18,19] - is functionally critical for immediate treatment success, whereas a subsequent upregulation of 'classical' language areas mediates long-term stability of intensive language training.

## Methods

**Figure 1:**
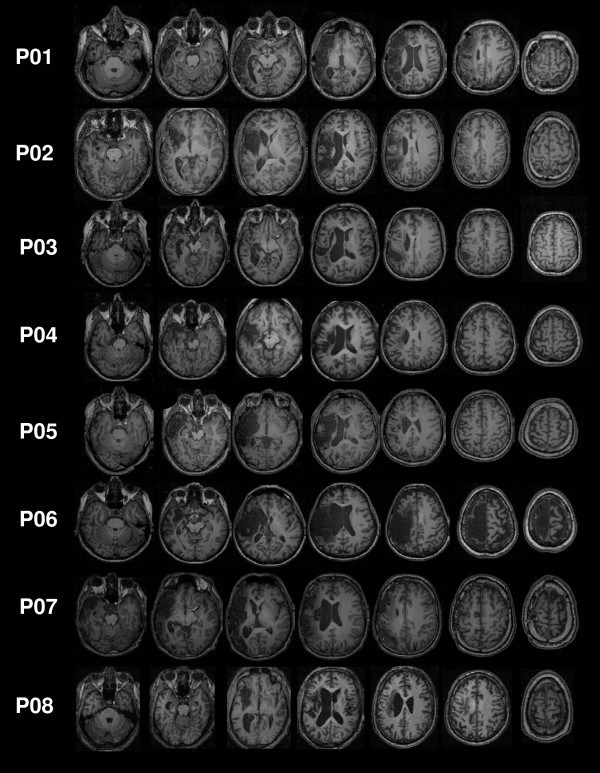
**Lesions**. Series of axial T1 images showing the extents of left hemisphere lesions for the eight patients

Eight patients (3 women; age: 34 to 67 years) with chronic aphasia and moderate to severe word finding difficulties (anomia) were studied. Detailed clinical and demographic information of the patients are provided in Additional file 1. Figure 1 shows the lesions of the patients. All patients suffered from anomia following a single left hemisphere ischemic stroke (except P03: hemorrhagic insult). The local Institutional Review Board had approved the study, written informed consent was obtained from all subjects and the study was conducted in accordance with the Helsinki Declaration.

Prior to training, all patients completed a baseline assessment consisting of a neurological examination, speech and language tests, and neuropsychological testing. All patients had relatively preserved cognitive abilities except for the language domain (see Additional file 2 for details).

Additionally, nine healthy control subjects (matched for age, sex, and handedness with the patients; 36 to 64 years old; 3 women) were examined twice within a two-week time interval on an overt naming task during fMRI to (a) establish the set of brain regions activated by healthy subjects during our naming task and (b) to control for effects of repeated testing [28].

### Anomia treatment

For the intensive anomia training, 50 concrete object names were individually selected for each patient ('trained objects'). Each trained object name had been named incorrectly at least twice during three baseline runs comprising a standardized set of 344 objects [29] (one correct run during baseline and post assessments was considered a random performance fluctuation). Patients received three hours of computer-assisted naming therapy per day over a period of two weeks. Each of the 50 trained objects occurred with a high repetition rate on each training day (up to 32 presentations) to promote stable long-term memory consolidation. Previously, it has been shown that this training approach is highly effective to improve word-retrieval in chronic aphasia patients [29]. Patients received three hours of daily computer-assisted naming treatment over a period of two weeks. The training comprised an associative language learning procedure that aimed at strengthening semantic associations between target words (object pictures) and auditory and graphemic cues to facilitate word-retrieval and followed the method of 'vanishing cues' [30] with five difficulty levels. At the first level, a picture of an object in combination with its spoken and written word form was presented, and the patient was asked to repeat the object name. Each training block comprised 200 object pictures (50 objects times 4 different object tokens). Training blocks on level 1 were repeated until the patient scored >80% correct responses. At the second difficulty level, cues were reduced so that the object picture was only cued with the first two phonemes and the first two graphemes (instead of the entire object name). At the third level, phonological and graphemic cues were reduced to the first phoneme. At level four, only the first grapheme was presented as a cue. Object naming without phonological or graphemic cues was required at the fifth level ('free naming'). Whenever performance was lower than 80 percent correct on levels two to five, a training block of level one was interspersed to provide patients with the complete visual and auditory target word forms. Patients were not provided online feedback during a block (except when perseverative naming errors occurred), but were informed about their overall scores after each training block. The computer-assisted training was supervised by an experienced speech and language therapist, who also scored each patient's response with yes or no using a keyboard connected to a PC. The language therapist was not involved in the assessment of primary and secondary outcome measures or in data analysis.

A separate set of 30 object names matched for several linguistic parameters (name agreement, word frequency, word-length, semantic categories, visual complexity of the pictures) served as a control set for the post assessments (immediately and eight months post training). During the post assessments, the 50 trained and 30 untrained objects were presented thrice outside the scanner.

Performance for a respective object name was classified as correct when at least two of the three runs were correct (i.e., perfect productions). By definition, the baselines for trained and untrained object names were zero percent correct (one or less correct runs out of three for each of the object names).

### MRI protocol

MRI data were acquired in a 3 Tesla whole body scanner (Philips Intera T30) with nominal gradient strength 30 mT/m and maximal slew rate 150 mT/m/ms. A circularly polarized transmit/receive birdcage head coil with a HF reflecting screen at the cranial end was used for spin excitation and resonance signal acquisition. Functional images were acquired using a T_2_* weighted single shot echo-planar (EPI) sequence (whole brain coverage, 36 transversal slices orientated parallel to the AC-PC line, slice thickness 3.6 mm without gap, FOV 230 mm, reconstruction matrix 64 × 64, in-plane resolution 3.6 × 3.6 mm^2^, i.e. isotropic voxels with 3.6 mm edge length, TE = 40 ms, TR = 3000 ms, flip angle = 90°). A high-resolution T1-weighted anatomical image was acquired for anatomical identification and coregistration into the Talairach space.

### fMRI task

The object-naming task comprised the visual presentation of photos of concrete objects during fMRI. Patients attended three identical fMRI sessions: The first was prior to the training (pre), the second directly after the training (post1), and the third eight months after completion of training (post2). Please note that patient P06 did not attend the 'two weeks'-appointment due to health problems unrelated to the study. The 'eight months'-assessment for P08 could not be conducted due to a required scanner hardware upgrade after his initial training.

**Figure 2:**
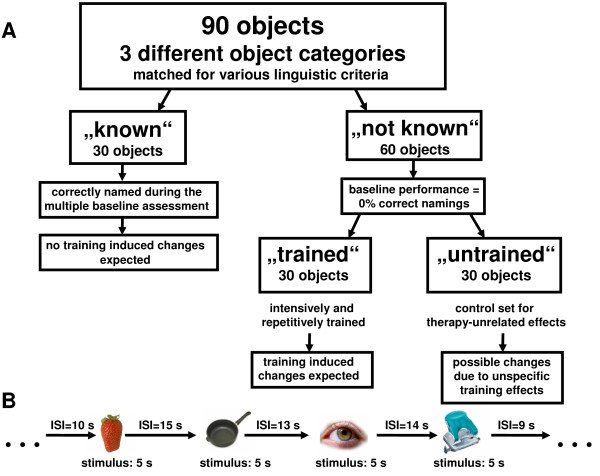
**Study design**. Flowcharts A: explaining the different object categories used during fMRI and B: illustrating the fMRI paradigm. ISI = Interstimulus interval

During each fMRI session, the participants had to overtly name the same 90 individually selected objects (Figure 2A). Thirty of the 90 objects had been consistently correctly named during the multiple baseline assessments. Half of the 60 incorrectly named objects at baseline were trained during the intensive therapy (a subset of the total 50 trained object names). The remaining 30 of the incorrectly named objects at baseline served as an untrained control set. Object names of the three different categories were matched for various linguistic criteria and were presented in random order during each of the three sessions (but fixed order for the three sessions per patient). Prior to scanning, the naming task was trained with all patients (using different objects) outside the scanner.

Naming performance was assessed during the respective fMRI session by means of a special MR dual-channel communication system, equipped with software to subtract gradient noise from the patient microphone channel (MR Confon GmbH, ). Control subjects also overtly named ninety visually presented objects during fMRI that were selected from our standardized set of 344 object pictures, based on a name agreement of > 80 percent in a different sample of healthy adults [29].

Images were projected using the stimulation software Presentation^® ^onto a screen fixed at the rear opening of the MR bore, which could be seen by the participant via a mirror fixed on the top of the head coil. Each of the 90 objects was presented for 5 s, followed by variable inter-stimulus intervals (ISI; 8-16 s, mean 12 s, Figure 2B). Subjects were instructed to overtly name the shown object during the 5 s presentation interval.

We used an event-related design proposed by Birn et al. [31] for fMRI studies involving overt verbal responses. Comparing different designs and analysis strategies, optimal detection of BOLD signal changes without significant motion artefacts were found for minimum stimulus durations (SD) of 5 s and average ISIs of at least 10 s.

### fMRI data analysis

Imaging data were analysed with SPM2 . Scans of each individual were corrected for slice timing and then realigned to the first session image. The functional images were first co-registered to the individual high-resolution anatomical scan. Then, the anatomical image was used to determine the parameters for the spatial normalization process [32]. During normalization, cost-function masking was performed to prevent distortion of the image due to the lesion [33]. The mask excluded the lesioned area and highly extended (ex vacuo dilated) ventricles. The resulting voxel size in standard stereotactic coordinates was 3 × 3 × 3 mm^3^. The normalized images were spatially smoothed using an isotropic Gaussian kernel (FWHM 9 mm).

Data were analysed in the context of the general linear model, using the canonical hemodynamic response function (hrf) to model responses during the five seconds of overt object naming for each experimental condition. To account for movement artefacts, the estimated realignment parameters were entered in the design matrix as user specified regressors. Anatomical localization of activated brain regions was determined using the Talairach Demon [34].

Planned contrasts-of-interest were calculated for each individual subject (first level analysis). These included the individual activity changes from pre to post training (post1-pre) or the comparison of the baseline scan with the third scan eight months after the training (post2-pre) for each object type separately (i.e., 30 trained objects, 30 untrained objects, 30 objects named correctly during the baseline assessment). To detect areas with the greatest short- or long-term fMRI activity changes, which linearly correlated with behavioural improvement, we performed two linear regression analyses (second level analysis). Dependent variable was the individual activity change from pre to post training (post1-pre, post2-pre), respectively. The respective individual training success for trained items (post1 or post2) served as behavioural regressor.

Technical difficulties, which could not be resolved by the fMRI microphone manufacturer, precluded the recording of the speech samples during scanning for later off-line analysis with application of a software filter to extract the speech signal from the scanner noise. Online assessment of patient's word production was not reliably possible because of the scanner background noise. Sound quality was, however, good enough to determine that all patients complied with task instructions and tried to name the objects presented during scanning.

Thus, we decided to use the patients' performance outside of the scanner on the matched set of items (here, each object was presented three times) as behavioural regressors. Even though intra-scanner scores are considered to be the most appropriate measure of performance for language production tasks [35], our extra-scanner measure assessed on the same day provides an appropriate estimate of the patients' performance. Furthermore multiple baseline assessments (here 3 runs per object name) represent a far more reliable performance measure in patients with aphasia with inherent performance fluctuations [36].

To ensure that the results are specific for overt naming of the trained objects and not due to therapy-unrelated activity changes over time (e.g., effects of repeated exposure, increased motor activity), we used the results of the respective regression analyses for the matched set of untrained objects as exclusive masks (i.e., areas correlated with unspecific activity changes were 'masked out'). The behavioural regressor here was the performance improvement of each patient from pretraining to post1 or post2, respectively. Thus, this analysis allows assessing which areas are specifically related to training-induced improved word-retrieval in the patient group.

To determine the brain activity pattern during overt picture naming in the nine healthy subjects, we performed a one sample t-test using the individual fMRI activation patterns during overt object naming (first fMRI session only). The nine healthy control subjects showed overt naming related activity in brain regions mediating the core processes of picture naming [37] [for details of the activity patterns of the healthy adults see Additional file 2]. Unspecific effects of repeated assessments were quantified by comparisons between sessions (Session 1 > Session 2 and vice versa) using paired t-tests. Activity patterns of the healthy controls at the first session as well as of the patients at each of the three assessments are presented in additional file 2.

Maximally activated voxels within significant clusters (p < 0.05) are reported at a voxel threshold of p < 0.05 and a minimum cluster size of 10 voxels if not otherwise stated. At least one voxel within a given cluster had to additionally survive a single-voxel statistical threshold of p < 0.001 (*Z-*score > 3.00) for the patients because of the known stronger activations seen in chronic stroke patients compared to healthy controls in fMRI studies [38]. Please note, for the present data analyses we applied a statistical threshold that does not correct for multiple comparisons, which protects against detection of false positives. Still, unlike simple t-tests, the regression analyses we used require close (linear) relationships between brain activity patterns and behavioural performance. Additionally, we exclusively masked the results with those of a parallel set of untrained object names to control for treatment- unrelated effects. This greatly reduces the risk to obtain false positives. Moreover, the substantial correlations between behavioural performance and activity changes within detected clusters (see results) were clearly not outlier-driven.

## Results

### Behavioural results

**Figure 3:**
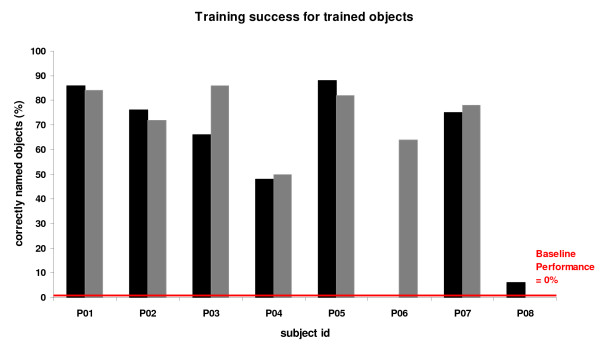
**Behavioural results for trained object names**. Training success for *trained *object names after two weeks (black) and after eight months (grey) for the eight patients. Please note that patient P06 did not attend the 'two weeks'-appointment due to health problems unrelated to the study. The 'eight months'-assessment for P08 could not be conducted due to a required scanner hardware upgrade after his initial training

**Figure 4:**
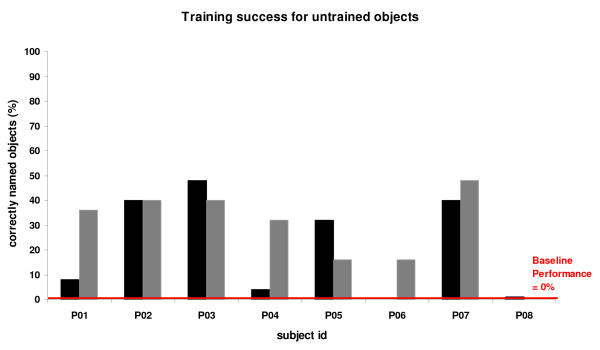
**Behavioural results for untrained object names**. Behavioural changes in naming performance for *untrained *object names after two weeks (black) and after eight months (grey) for the eight patients. Please note that patient P06 did not attend the 'two weeks'-appointment due to health problems. The 'eight months'-assessment for P08 could not be conducted due to a required scanner hardware upgrade after his initial training

On average, patients' performance improved during training, i.e., from zero percent correct naming responses at the baseline assessment to a mean of 64.4 +/- SD 26.7 percent correct responses immediately after training (details of improved naming of trained object names are shown in Figure 3). Performance for untrained object names also improved from baseline to immediate post training assessment, but to a lesser degree (mean of 25.0 +/- 18.5 percent correct) (see Figure 4). This training-unspecific improvement may be either due to the repeated presentations of untrained objects over the course of the three assessments or an improved general word retrieval strategy after training. Behavioural improvement (as compared to the baseline) for the trained objects was significantly greater for trained as compared to untrained object names (Object name set × Session: F(2,14) = 19.30, 0.001, Greenhouse-Geisser corrected). This was verified in posthoc analyses for both the immediate as well as the 8-month follow-up assessments (both F(2,14) ≥ 25.56, both p < 0.001, Greenhouse-Geisser corrected). The effect size for short-term success (based on the mean difference between trained and untrained object names) amounted to Cohen's *d *= 1.7, which is a large treatment effect. Performance remained highly stable at the eight months post training assessment (see Figure 3). Analysis of the error distributions revealed that semantic paraphasias were the most common error type at both the baseline and the two post assessments for all patients (main effects of error type: all F(5,30/5,35) > 3.40, p < 0.01), and training resulted in a comparable improvement for all error types (see Additional file 2).

### fMRI results aphasia patients

**Figure 5:**
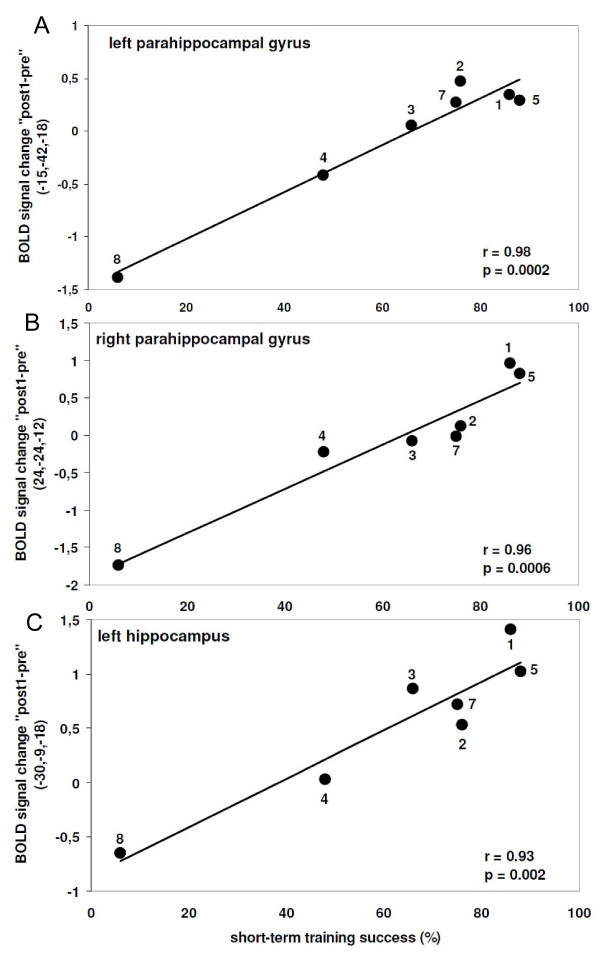
**Results of the regression analyis for trained items immediately after training**. Positive correlation between short-term training success and 'post1 - pre' training activity changes for trained object names in memory related structures (A) the left parahippocampal gyrus (B) the right parahippocampal gyrus and (C) the left hippocampus for the group of aphasia patients [Note: signal change refers to the peak voxel within significant clusters]

Greater *short-term *treatment success was related to *increased *activity (see Additional file 3a) bilaterally in the parahippocampi (Figure 5A + B; left parahippocampus: r(5) = .98, p = .0002; right parahippocampus: r(5) = .96, p = .0006) and in the left hippocampus (Figure 5C; r(5) = .93, p = .002). Please note, removing the data of the patient who suffered from global aphasia and did not benefit from the training (P08) did not substantially affect the significant correlations [left parahippocampal gyrus: r(4) = .88, p = .019; right parahippocampal gyrus: r(4) = .84, p = .034, left hippocampus: r(4) = .84, p = .033]. Additionally, activity increases in the right parietal cortex (precuneus, BA 7), the right cingulate gyrus (BA 31), and bilaterally in the occipital lobes (BAs 19/37) were correlated with behavioral improvement. There were no brain regions for which *decreases *of activity correlated with treatment success immediately after the training (see Additional file 2b).

**Figure 6:**
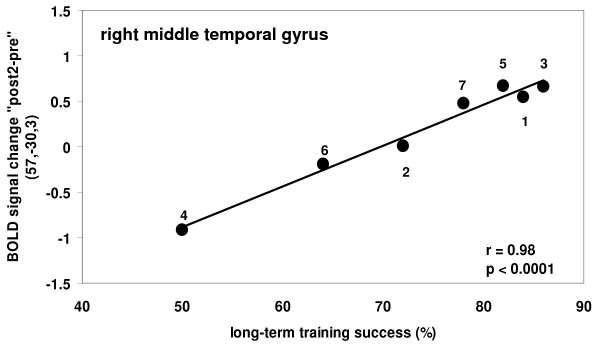
**Results of the regression analyis for the follow-up assessment**. Positive correlation between long-term training success and 'post2 - pre' training activity changes for trained object names in the right middle temporal gyrus for the group of aphasia patients (signal change refers to the peak voxel within significant clusters)

*Long-term *success for trained object names (see Additional file 2c and 2d) was associated with the degree of activity *increase *in the right-sided Wernicke's homologue (BAs 21/22; Figure 6; r(5) = .98, p < .0001). Additionally, increased left perilesional middle and superior temporal areas (BA 21; x/y/z: -51/3/-12; r(5) = .80, p = .03) activities were associated with training success at a less stringent correction level (Z = 2.17). Greater long-term treatment success was also related to stronger activity *decreases*, suggestive of greater automation, in left supplementary motor areas (BA 6), right inferior parietal (BA 40) regions, the right fusiform gyrus (BA 19), and the right caudate nucleus.

In healthy control subjects, the comparison between the two fMRI sessions revealed no *increases *of brain activity ('Session2 > Session1'), even at an uncorrected threshold of p < 0.05. Only activity *decreases *in the right inferior parietal lobe were observed from the first to the second assessment (BA 40; p < 0.005 voxel threshold, p < 0.05 cluster threshold).

## Discussion

The present fMRI study determined for the first time both short- and long-term correlates of successful treatment-induced language recovery in chronic aphasia. We here show that success of an intensive language training in chronic aphasia patients depends both on immediate activity changes in visual, attention-, and memory-related brain structures as well as on slowly evolving activity changes (over the course of eight months) in right language homologue areas of the middle temporal lobe (and to a lesser extent in perilesional temporal areas). This longitudinal study thus demonstrates that even in the chronic stage after a stroke, language reorganization is a highly dynamic process, comparable to the time course of language or motor recovery in the acute stage after stroke [9,38].

### Short-term treatment success

Patients behaviourally improved from zero percent correct naming responses at pre-training to a mean of 64.4 percent immediately after training, which amounts to a very large treatment effect compared to previous studies with chronic aphasia patients [39]. This demonstrates that an intensive behavioural training based on associative learning principles, coupled with a high stimulus repetition rate, can be highly effective in chronic aphasia.

Immediate treatment success from baseline to immediately after training was best predicted by an fMRI activity increase in brain structures orchestrating memory encoding (left hippocampus, bilateral parahippocampi) [21]. It may seem surprising at first, that memory-related areas are predominantly involved in the initial recovery of overt picture naming. However, an association between aphasia therapy outcome and the functional integrity of memory-related brain structures has been noted previously. Acute aphasia patients 'with lesions to temporobasal regions showed less improvement during therapy and less total recovery, but a similar amount of spontaneous recovery than patients without such lesions' [40] (p. 684). The authors interpreted this finding as evidence for a disconnection of the hippocampus and perisylvian language areas in patients with poor (intensive) training outcomes, which should be targeted in future studies using diffusion-tensor imaging. Furthermore, several recent functional imaging studies in healthy subjects have shown that the (left) hippocampus contributes to *initial *language learning, i.e., the acquisition of lexical [25], semantic [26], and syntactic [27] knowledge, and left hippocampal activity during vocabulary learning was an excellent predictor of vocabulary efficiency post training [25]. The convergence of findings in aphasia patients and healthy subjects points to comparable neural mechanisms during language acquisition in neurologically intact adult subjects and post-stroke language recovery [38]. Thus, in the future, a miniature treatment session with functional imaging as part of the baseline assessment may allow to determine the functional integrity of the language encoding neural network in order to predict the likelihood of subsequent treatment success.

Additionally, a right parahippocampal activity increase from pre to post training was a good correlate of language recovery in our patients. This presumably reflects the fact that language re-learning is more laborious in aphasia as compared to language learning in healthy subjects and may thus require the joint effort of both hemispheres. The right parahippocampus may also mediate the functional recruitment of right-sided homologue language regions following left hemisphere damage [23,24].

We also observed increased activity immediately after training in the right-sided precuneus and cingulate gyrus and bilaterally in the posterior fusiform gyri in patients with good naming recovery. This in line with previous functional imaging studies on treatment induced recovery of language functions in chronic aphasia [11] and these areas are known to mediate attention and initial cross-modal integration of visual, phonological, and semantic information [41].

A lack of treatment-induced hippocampal activity modulation in prior aphasia intervention neuroimaging studies may be due to the smaller extent of treatment induced language recovery in chronic aphasia (e.g. [5,18,19]). A visual inspection of our fMRI data revealed that activity increases in the left hippocampus only occurred for the four patients who scored 75 percent correct naming responses and higher immediately after training. This implies that activity increases in the hippocampal formation are only observed in patients with very high treatment gains. A different explanation might be that our findings can be explained by the training paradigm we used in this study that strongly relies on associative memory performance and requires the hippocampus. This issue has to be clarified in future group studies employing different types of language training paradigms (e.g., [17,42]).

### Long-term treatment success

The major novel aspect of the present study was the identification of brain structures mediating the long-term retention (after eight months) of the training outcome. Behaviourally, patients' naming performance remained highly stable throughout the eight months follow-up period (correlation between short- and long-term training successes: r = 0.76, p < 0.05). The long-term treatment success, however, was mediated by different brain structures than those involved in short-term language recovery. Greater treatment success in the long run was predominantly related to an activity increase in language regions of right temporal lobe (i.e., the right-sided Wernicke's homologue; BAs 21,22) - when lowering the statistical threshold to account for the reduced statistical power in perilesional brain areas [43] - in perilesional middle temporal areas. The predominance of increased functional activity in right-temporal areas might be explained by the large cortical-subcortical lesions in our patient sample and the large intersubject variance in terms of lesion location and extent. There is evidence from other studies with more limited and homogenous lesions [e.g., [19,44,45]] that better recovery is mainly associated with activity in spared left perilesional areas. On the other hand, middle and superior temporal lobe regions in both hemispheres are known to be important for the reliable retrieval of lexical-semantic knowledge in healthy subjects [37,44,45]. Thus, when lesions are large and the residual linguistic deficit is substantial (as was the case in most of our participants), right temporal regions may contribute to effective functional compensation. Furthermore, in acute aphasic patients, the degree of *spontaneous *language recovery over the course of one year depended on regional cerebral blood flow increases bilaterally in the temporal lobes [46,47]. The activity increase in our patients can thus be interpreted as reflecting strengthened neural connections between the semantic representations of the objects and their respective word forms as a result of the intensive anomia training. Therefore, language areas in the temporal lobe of both hemispheres seem to subserve long-term language therapy success. At the eight months post assessment, negative correlations with training success were found for the right inferior parietal cortex (BA 40), right occipital areas (BA 19) and for right motor related areas (BA 6, caudate nucleus), possibly related to greater automation, similar to the decreased activity found in healthy controls subjects after repeated testing.

### Limitations of the current study

The current study aimed to investigate the neural correlates of immediate versus long-term successes of intensive naming training in chronic aphasia patients. The results can therefore not be directly generalized to other types of language training (non-intensive) or other types of aphasic symptoms (e.g., patients with predominantly phonological paraphasias). Future studies may also consider obtaining complimentary information from different imaging techniques that have a better temporal resolution. For example, Laganaro et al. [48] used electroencephalography (EEG) and provided evidence for distinct abnormalities in the time-course of evoked potentials for different types of anomia.

Furthermore, the core problem in our patients was the linking of semantic information with a particular word form (see Additional file 2). DeLeon et al. [49] reported that the degree of lexical-semantic impairment correlated with hypoperfusion in left BA 22 in the acute stage after ischemic stroke. The slowly evolving activity changes in the superior and middle temporal lobes as a result of our training may thus be specific for the successful recovery of lexical access and not for other production processes involved in picture naming (e.g., syllabification or phonetic/articulatory preparation). This might also be an explanation for the fact that we did not find activity increases in right or left (inferior) frontal (IFG) brain areas that have been linked to improved word-retrieval in previous treatment studies assessing overt language production (for review see [11]). In particular, left inferior frontal activity during picture naming has been linked to effortful word retrieval [50], while highly overlearned materials (like the items in our study) do not necessarily elicit activity in this brain area [28]. In line with these previous findings, in our own study, (right) IFG activity was only found during the first imaging session but not immediately after the intensive treatment period (see additional file 2 for details of activity patterns elicited by trained pictures at the pre-training assessment).

## Conclusion

Our findings suggest that different brain regions are required for initial learning versus long-term consolidation induced by intensive training in chronic aphasia. For initial learning, activity increases bilaterally in brain regions involved in memory, attention, and multimodal integration predicted treatment success. The long-term treatment success, however, was particularly mediated by activity increases in the right-sided Wernicke's homologue and left temporal language areas, suggesting bilateral recruitment of 'normal' task-related areas during the consolidation process. The results show that language recovery induced by intensive training is a dynamic, slowly evolving process involving both hemispheres requiring both classical language areas and domain-unspecific attention and memory brain structures - even in the chronic stage after stroke.

## Authors' contributions

RM and MM contributed equally to the study (shared first authorship). RM, MM, HK, MD, AB, HS, MT, KK, HL, AF, SK and CB contributed to the design of the study. RM, MM, HK, HS, KK and CB collected the imaging data. HL conducted the neuropsychological testing. RM, KK and MT administered the treatment. RM, MM, HK, MD and CB analyzed the fMRI data. RM, MM and CB drafted the manuscript. All authors were involved in revising the initial draft and approved the final version.

## Supplementary Material

Additional file 1 **Patient Information**. Demographic and clinical information and language test results for the eight aphasia patients Click here for file

Additional file 2 **Supporting information**. Provides additional supportive information regarding patient characteristics, neuropsychological testing and functional imaging analyses and results Click here for file

Additional file 3 **Brain activity changes immediately after training and at the follow-up assessment**. Brain activity changes in the patients and healthy controls: Brain areas showing a) positive and b) negative correlations between short-term training success and 'post1-pre' training activity changes for trained object names (masked with the respective results for untrained object names). Brain areas showing c) positive and d) negative correlations between long-term training success and 'post2-pre' training activity changes for trained object names (masked with the respective results for untrained object names). The corresponding results for the control group (Session1 versus Sesssion2) are displayed in italic font Click here for file
