# Supplementary material for: Imaging short- and long-term training success in chronic aphasia
Source: BMC Neurosci. 2009 Sep 22;10:118. doi: 10.1186/1471-2202-10-118 (PMC2754483; doi:10.1186/1471-2202-10-118)
Supplement: Additional file 1 — Patient Information. Demographic and clinical information and language test results for the eight aphasia patients [file 1471-2202-10-118-S1.DOC]

**Additional file 1**

| **Patient** | **P01** | **P02** | **P03** | **P04** | **P05** | **P06** | **P07** | **P08** |
| --- | --- | --- | --- | --- | --- | --- | --- | --- |
| **Age (years)** | 34 | 47 | 55 | 53 | 45 | 55 | 43 | 67 |
| **Sex (m=male, f=female)** | m | m | f | F | m | f | m | m |
| **Years post stroke** | 6.92 | 4.92 | 4.75 | 6.00 | 5.08 | 5.75 | 1.83 | 4.45 |
| **Handedness (Edinburgh Handedness Inventory)** | 80 | 80 | 100 | 100 | 30 | 50 | 100 | 100 |
| **Aachen Aphasia Test (AAT)** |  |  |  |  |  |  |  |  |
| Spontaneous speech scales * [scores, max. 5] | 2-3-2-2-2-1 | 2-4-4-3-2-1 | 1-2-2-3-2-1 | 2-3-2-2-3-2 | 2-4-4-3-4-2 | 2-5-4-4-5-1 | 1-3-5-3-2-1 | 1-2-2-3-4-1 |
| Token Test [error score, max. 50/ PR] | 50/ 2 | 40/ 28 | 37/ 33 | 42/ 22 | 47/ 10 | 31/ 44 | 39/ 30 | 30/ 46 |
| Repetition [raw score, max. 150/ PR] | 59/ 21 | 120/ 60 | 99/ 41 | 109/ 48 | 112/ 51 | 121/ 61 | 108/ 48 | 134/ 77 |
| Written language [raw score, max. 90/ PR] | 68/ 70 | 52/ 53 | 30/ 36 | 29/ 36 | 24/ 32 | 24/ 32 | 54/ 56 | 37/ 42 |
| Naming [raw score, max. 120/ PR] | 34/ 28 | 79/ 51 | 59/ 40 | 74/ 47 | 97/ 75 | 70/ 44 | 89/ 62 | 25/ 22 |
| Language Comprehension [raw score, max. 120/ PR] | 84/ 58 | 94/ 73 | 83/ 56 | 74/ 47 | 67/ 35 | 77/ 47 | 89/ 65 | 71/ 39 |
| syndrome classification | moderate to severe Broca´s Aphasia | moderate to severe Broca´s Aphasia | severe Broca’s aphasia | moderate to severe Broca´s Aphasia | moderate to severe Broca´s Aphasia | moderate to severe Broca´s Aphasia | moderate to severe Broca´s Aphasia | Severe  global  aphasia |

AAT = Aachen Aphasia Test

* rating scales for spontaneous speech are (min. score of 0 and max. score of 5): communication, articulation, speech automatisms, semantic, phonematic, syntax

PR = percent rank.
