# Supplementary material for: Imaging short- and long-term training success in chronic aphasia
Source: BMC Neurosci. 2009 Sep 22;10:118. doi: 10.1186/1471-2202-10-118 (PMC2754483; doi:10.1186/1471-2202-10-118)
Supplement: Additional file 2 — Supporting information. Provides additional supportive information regarding patient characteristics, neuropsychological testing and functional imaging analyses and results [file 1471-2202-10-118-S2.DOC]

**Additional file 2**

**Additional supporting information**

**Patient characteristics and neuropsychological testing**

A group of eight chronic aphasia patients was recruited for the study (all > 12 months post onset, all suffering from Broca’s aphasia except one patient (Global aphasia), all word finding difficulties with predominantly semantic paraphasias). Inclusion criteria for the patients comprised: no MR contraindications, no severe apraxia of speech as assessed by the Bogenhausener Dysarthrie Skalen [1], premorbid right-handedness, German as native language, no serious impairment of vision or hearing, relatively preserved comprehension of instructions as determined by clinical assessment, no signs of dementia (as assessed by the Mini Mental Status Test [2]) or depression (as assessed by the Visual Analogue Mood Scales [3]), no psychiatric or additional neurological diagnoses or other serious medical conditions.

Prior to training, all patients completed a baseline assessment consisting of a neurological examination, speech and language tests, and neuropsychological testing. The baseline speech and language assessments comprised an auditory screening for hearing ability, assessment of apraxia of speech [1], auditory discrimination tasks (pitch, speech rate, words, nonwords [4]) and the Aachen Aphasia Test, AAT [5]). As part of the baseline language assessment, patients also completed an object naming task split over three days, during which a standardized set of 344 different objects [6] had to be named thrice. Accuracy of the first response was scored online by a certified speech therapist (verified by offline analysis). Error categories included semantic paraphasias, phonemic paraphasias, mixed semantic and phonemic paraphasias, unrelated errors, neologisms (new word creations without meaning), and non-reactions (see Tables 1+2 for error distributions of the baseline assessment and during the three testing sessions prior to and after training and at the follow-up assessment). The naming errors in our patient sample revealed an inconsistent pattern across the repeated assessments (e.g., the picture of a horse was named ‘deer’, ‘cow’ or ‘sheep’), which is in line with underspecified lexical-semantic information or impaired selection processes [7]. Also, during the treatment sessions, the subjects responded well to phonological cues, which is rarely observed in patients with amodal semantic deficits (e.g., semantic dementia [7]). Moreover, neither the clinical impression nor the relatively preserved speech-comprehension subtests of the AAT indicated a gross deficit in amodal semantic knowledge. Only a small fraction of the errors in our patient sample were phonemic paraphasias (0-10%) and repetition of single words was relatively preserved (AAT-repeating subtest). This pattern of errors indicates that the patients’ main deficit was related to a selective impairment of lexical-semantics (i.e., the linking of semantic information with a particular word form [8]).

The neuropsychological test battery comprised the assessment of premorbid handedness (Edinburgh Handedness Inventory) [9], associative learning of a miniature lexicon [10] (data not reported here), short- and long-term memory (subtests ‘Digit Span’ and ‘Visual Memory Span’ (Corsi block tapping) of the German Wechsler Memory Scale - Revised (WMS – R [11])), Rey Complex Figure Test [12] (RCFT); Benton test [13]), general learning capacity (subtests ‘verbal’ and ‘visual paired associates’ of the WMS – R [11]), phonemic and semantic word fluency (subtests from the verbal-fluency-test [14]), general intellectual functioning (subtests ‘picture completion’ and ‘block design’ of the German Wechsler Adult Intelligence Scale (HAWIE-R [15]), attention and executive functions (Trail Making Tests A and B [16]) and the modified Wisconsin Card Sorting Test [17] (see Table 3).

**Functional magnetic resonance imaging**

Nine healthy subjects were scanned twice within a two week interval with the same naming task during fMRI. To determine the brain activity pattern during overt picture naming in the nine healthy subjects, we performed a one sample t-test using the individual fMRI activation patterns during overt object naming (first fMRI session only, p< 0.001).

To determine the brain activity pattern during overt picture naming for the trained objects in aphasia patients, we performed one-sample t-tests using the individual fMRI activation patterns during the 5 s of overt object naming for each of the three different time points (p< 0.01).

Additional analyses aimed to find commonalities in brain activity during overt naming in healthy subjects and aphasia patients with good treatment response, i.e., we assessed whether increased activity was located within the brain network activated by picture naming in the healthy control subjects. For this purpose, we performed simple linear regression analyses as implemented in SPM2. Dependent variable was the individual fMRI activity change from pre to post training in the aphasia patients (‘post1-pre’ or ‘post2-pre’, respectively). The respective individual training success for trained items (post1 or post2, analyses included all 30 responses) served as regressor. An inclusive mask (activity was set to ‘one’ within the mask), based on the results of the one sample t-test for healthy subjects described above, was applied to the results of these regression analyses. Only clusters exceeding a single-voxel statistical threshold of p < 0.05 for a minimum of 10 voxels are reported.

**Results**

fMRI results

*Brain areas activated during overt object naming in healthy control subjects (Table 4)*

The nine healthy control subjects showed overt naming related activity in the left superior temporal and inferior frontal gyri (BAs 22/44), in the left anterior cingulate gyrus (BA 25), in the left superior parietal gyrus (BA 7), in the left primary motor cortex (BA 4) and bilaterally in the occipital lobe (BAs 18/19). Furthermore, the control subjects activated the right inferior frontal/superior temporal gyri (BAs 44/47/38), the right superior frontal gyrus (BA 6/10), the right middle temporal gyrus (BA37), the right inferior (BAs 40/2) and superior parietal cortex (BAs 7/19), the right cuneus (BA 18), and the right thalamus, putamen, pallidum, and the parahippocampal gyrus (BAs 34/35).

*Brain areas activated during overt naming of trained objects in patients (Table 5):*

*i) Prior to training (‘pre’)*

At the baseline assessment, overt naming related activity was found in the right inferior frontal gyrus (BAs 47), the right supplementary motor area and insula (BAs 6/13), and the right posterior cingulate gyrus (BA 29). Furthermore, prior to training, all patients activated the left parahippocampal gyrus (BA 30) and the occipital cortex bilaterally (BA 19).

*ii) Immediately after intense training (‘post1’)*

The seven patients who attended the ‘post1’ assessment (P01-P05 and P07-P08) showed overt naming related activity in the right primary motor cortex (BA 4), the right middle and superior temporal gyrus (BAs 21/22), the right medial frontal gyrus (BAs 6/32), and the right posterior cingulate cortex (BA 30). Furthermore, the analysis revealed activity in the left and right occipital lobe (left: BAs 18/19; right: BAs 37/20).

*iii) Eight months after intense training (‘post2’)*

Eight months after training ( ‘post2’ assessment) overt naming related activity was found in the right inferior frontal/anterior superior temporal gyri (BAs 47/22), in the right insula and supplementary motor area (BAs 13/6), and in the right posterior cingulate gyrus (BA 29). Additional activity was found in the occipital lobe of both hemispheres, with peak activity located in the right BA 19.

*Training induced changes in brain activity within regions activated in healthy subjects*

Short-term treatment success. Activity *increases* from pre to immediately post intense anomia training were observed in two brain regions which were also activated by healthy subjects during overt object naming. These comprised the right parahippocampal gyrus/hippocampus (BA 28/35, MNI-coordinates: 24, -24, -12, Z = 3.45) and the right globus pallidus (MNI-coordinates: 21, -15, 3, Z = 3.69). Activity *decreases* from pre to post intensive anomia training were not seen in of the brain regions activated by the healthy subjects during overt naming.

Long-term treatment success. Activity *increases* from pre to 8 months post intensive anomia training overlapped with the activation pattern of the healthy subjects in the right middle temporal gyrus (BA 22, MNI-coordinates: 66, -30, -3, Z = 3.14) and in the right superior frontal gyrus (BA 10, MNI-coordinates: 27, 42, 24, Z = 3.85). Activity decreases from pre to 8 months post training in brain regions activated by healthy control subjects during overt naming were located in the left inferior frontal gyrus (BAs 47, MNI-coordinates: 45, 18, 3, Z=3.59), the right occipital lobe (fusiform gyrus, MNI-coordinates: -24, -71, -19, BA 19, Z=3.30), and the left precentral gyrus (BAs 6/43, MNI-coordinates: 48, -3, 15, Z=3.19).

**Discussion**

*Brain areas activated during overt confrontation picture naming in healthy control subjects*

The nine healthy control subjects showed overt naming related activity in brain regions mediating the core processes of picture naming [18]. These were areas subserving lead-in processes of object naming, like object recognition (parahippocampal gyrus), verbal working memory (left anterior cingulate gyrus, inferior frontal gyrus), and the representation of phonologic sequences (right inferior parietal gyrus) [10,19,20]. Core object naming related activity was observed in regions reflecting the conceptually driven selection of a given lexical item and the retrieval of the lexical word form (left superior temporal gyrus) [18]. Control subjects furthermore activated regions involved in syllabification of the object names (left: BAs 22/44, right: BAs 44/47/38) [18]. The additional activation of motor-related brain regions (left primary motor cortex, right putamen, right thalamus, right pallidum) is presumably a neural correlate of phonetic encoding and articulation.

*Brain areas activated during overt confrontation picture naming in patients with aphasia*

Prior to training, when naming performance was at floor, patients only activated brain regions involved in vision (left/right BA 19), attention, and object recognition (right posterior cingulate gyrus, left parahippocampal gyrus), effortful selection processes (right inferior frontal gyrus) and articulation (right supplementary motor area, right insula) [18].

Immediately after training, patients activated bilateral visual areas (BAs 18/19/20/37), the right primary motor areas (BA 4), and the anterior and posterior cingulate gyri (BAs 32/6 and BA 30). The latter may reflect improved episodic recognition of previously encountered information [21] Additionally, we now observed activity in areas related to lexical access and/or phonologic code retrieval in the right middle and superior temporal lobe (BAs 21/22) [18].

Eight months after training activity in the right superior temporal/inferior frontal gyri (BAs 22/47) and the right insula/supplementary motor area (BAs 13/6) presumably reflected successful syllabification and articulation of trained object names [18].

*Training induced changes in brain activity within regions activated in healthy subjects*

Training induced *short-term* activity changes within regions activated during overt object naming in healthy subjects were observed in brain regions mediating *memory* (right parahippocampal gyrus) and *articulation* (right globus pallidus). For *long-term* activity increases in aphasia patients, an overlap with the naming related activity in healthy subjects was found in right *language* (middle temporal gyrus) and *attention* (superior frontal gyrus) areas.

Decreases in attention (inferior frontal), visual and motor areas (fusiform gyrus, precentral gyrus) were presumably related to reduced frontal executive task demands with increasing task familiarity, automation of verbal responses, and increased response certainty [18].

**Conclusion**

Our results demonstrate that the overt naming paradigm used in the present fMRI study produced valid brain activation patterns comprising all relevant sub-processes of object naming in healthy subjects [18].

Prior to training, patients activated vision-, motor-, and attention-related brain regions. After successful training, patients show naming-related activity in language areas involved in syllabification and articulation of object names. Overlapping activity patterns of chronic aphasia patients and healthy subjects showed that language recovery due to intense training led to activity changes particularly in regions which are part of the ‘normal’ neural object naming network.

**References**

1. Nicola F, Vogel M, Ziegler W: **Bogenhausener Dysarthrie Skalen (BODYS)**. *Forum Logopädie* 2004, **2**:14-22.
2. Folstein MF, Folstein SE, McHugh PR: **"Mini-mental state". A practical method for grading the cognitive state of patients for the clinician**. *J Psychiatr Res* 1975, **12**:189-198.
3. Stern RA, Arruda JE, Hooper CR, Wolfner GD, Morey GD: **Visual analogue mood scales to measure internal mood state in neurologically impaired patients**. *Aphasiology* 1997, **11**:59-71.
4. Liephold M, Ziegler W, Brendel B: **Hierarchische Wortlisten: Ein Nachsprechtest fuer die Sprechdiagnostik**. Dortmund: Modernes Lernen; 2003.
5. Huber H, Poek K, Weniger D, Willmes K: **Aachener Aphasie Test**. Göttingen: Hogrefe; 1983.
6. Schomacher M, Baumgärtner A, Winter RJ, Lohmann H, Dobel C, Wedler K, Abel S, Knecht S, Breitenstein C: **Erste Ergebnisse zur Effektivität eines intensiven und hochfrequenten Benenn- und Konversationstrainings bei Aphasie**. *Forum Logopädie* 2006, **20**:22-28.
7. Jeffries E, Lambon-Ralph MA: **Semantic impairment in stroke aphasia versus semantic dementia: a case-series comparison.** *Brain* 2006, **129**:2132-2147.
8. DeLeon J, Gottesman RF, Kleinman JT, Newhart M, Davis C, Heidler-Gary J, Lee A, Hillis AE: **Neural regions essential for distinct cognitive processes underlying picture naming**. *Brain* 2007, **130**:1408-1422.
9. Oldfield RC: **The assessment of handedness: The Edinburgh inventory**. *Neuropsychologia* 1971, **9**:97-113.
10. Breitenstein C, Jansen A, Deppe M, Foerster AF, Sommer J, Wolbers T, Knecht S: **Hippocampus activity differentiates good from poor learners of a novel lexicon**. *Neuroimage* 2005, **25**:958-968.
11. Härting C, Markowitsch HJ, Neufeld H, Calabrese P, Dejerine J, Deisinger K, Kessler J: **Wechsler Gedächtnistest - Revidierte Fassung (WMS-R)**. 2000.
12. Meyers JE, Meyers KR: **Rey Complex Figure Test and Recognition Trial**. Lutz: Psychological Assessment Resources; 1995.
13. Benton AL: **The revised visual retention test**. Bern: Huber; 1981.
14. Aschenbrenner S, Tucha O, Lange KW: **Regensburger Wortflüssigkeitstest (RWT)**. Goettingen: Hogrefe; 2001.
15. Tewes U: **Hamburg Wechsler Intelligenztest für Erwachsene - Revision - HAWIE-R**. Bern: Huber; 1991.
16. Tombaugh TN: **Trail Making Test A and B: normative data stratified by age and education**. *Arch Clin Neuropsychol* 2004, **19**:203-214.
17. Nelson HE: **A modified card sorting test sensitive to frontal lobe defects**. *Cortex* 1976, **12**:313-324.
18. Indefrey P, Levelt WJ: **The spatial and temporal signatures of word production components**. *Cognition* 2004, **92**:101-144.
19. Paulesu E, Frith CD, Frackowiak RS: **The neural correlates of the verbal component of working memory**. *Nature* 1993, **362**:342-345.
20. Gelfand JR, Bookheimer SY: **Dissociating neural mechanisms of temporal sequencing and processing phonemes**. *Neuron* 2003, **38**:831-842.
21. Ries ML, Schmitz TW, Kawahara TN, Torgerson BM, Trivedi MA, Johnson SC: **Task-dependent posterior cingulate activation in mild cognitive impairment**. *Neuroimage* 2006, **29**:485-492.

**Table 1** Baseline performance of the eight aphasia patients (set of 344 objects).

| **Patient** | **P01** | **P02** | **P03** | **P04** | **P05** | **P06** | **P07** | **P08** |
| --- | --- | --- | --- | --- | --- | --- | --- | --- |
| **correctly named objects (%)** | 23 | 42 | 24 | 36 | 54 | 53 | 60 | 0 |
|  | | | | | | | | |
| **percentage of different error**  **types (sum correctly named objects + errors = 100%):** |  | | | | | | | |
| correctly named objects (%) | 23 | 42 | 24 | 36 | 54 | 53 | 60 | 0 |
| semantic paraphasias (%) | 37 | 34 | 13 | 32 | 33 | 24 | 18 | 0 |
| phonemic paraphasias (%) | 8 | 7 | 9 | 5 | 1 | 2 | 10 | 0 |
| mixed (sem. + phon.) errors (%) | 1 | 1 | 2 | 0 | 0 | 0 | 3 | 0 |
| unrelated errors (%) | 9 | 2 | 1 | 3 | 3 | 3 | 0 | 0 |
| neologisms (%) | 7 | 5 | 14 | 9 | 8 | 6 | 7 | 0 |
| non-reactions (%) | 15 | 9 | 15 | 15 | 1 | 12 | 2 | 100 |

‘correctly named’ = objects correctly named twice or thrice during three naming sessions

‘incorrectly named’ = objects correctly named once or not once during three naming sessions.

**Table 2** Naming performance of the eight aphasia patients for the fifty trained object names (performance prior to training was zero percent correct by definition).

| **Patient** | **P01** | **P02** | **P03** | **P04** | **P05** | **P06** | **P07** | **P08** |
| --- | --- | --- | --- | --- | --- | --- | --- | --- |
| **correctly named objects (%)** |  |  |  |  |  |  |  |  |
| pre | 0 | 0 | 0 | 0 | 0 | 0 | 0 | 0 |
| post1 | 86 | 76 | 66 | 48 | 88 | - | 75 | 6 |
| post2 | 84 | 72 | 86 | 50 | 82 | 64 | 78 | - |
|  | | | | | | | |  |
|  | **percentage of different error types**  **(sum = 100%)** | | | | | | | |
|  | **semantic paraphasias (%)** | | | | | | |  |
| pre | 53 | 57 | 20 | 58 | 74 | 48 | 55 | 0 |
| post1 | 21 | 68 | 29 | 40 | 47 | - | 65 | 0 |
| post2 | 44 | 57 | 29 | 38 | 48 | 42 | 60 | - |
|  | **phonemic paraphasias (%)** | | | | | | | |
| pre | 8 | 13 | 6 | 8 | 2 | 6 | 20 | 0 |
| post1 | 26 | 14 | 0 | 17 | 6 | - | 20 | 0 |
| post2 | 12 | 10 | 6 | 22 | 9 | 14 | 27 | - |
|  | **mixed errors (sem.+phon.) (%)** | | | | | | | |
| pre | 1 | 1 | 6 | 0 | 1 | 0 | 5 | 0 |
| post1 | 11 | 0 | 3 | 0 | 0 | - | 4 | 0 |
| post2 | 8 | 0 | 0 | 0 | 0 | 0 | 0 | - |
|  | **unrelated errors (%)** | | | | | | | |
| pre | 12 | 1 | 1 | 1 | 6 | 8 | 0 | 0 |
| post1 | 37 | 0 | 3 | 2 | 18 | - | 0 | 0 |
| post2 | 8 | 3 | 6 | 6 | 13 | 0 | 0 | - |
|  | **neologisms (%)** | | | | | | | |
| pre | 8 | 9 | 16 | 14 | 16 | 13 | 19 | 0 |
| post1 | 0 | 14 | 26 | 28 | 29 | - | 15 | 0 |
| post2 | 28 | 13 | 29 | 24 | 22 | 11 | 10 | - |
|  | **non-reactions (%)** | | | | | | | |
| pre | 18 | 19 | 51 | 19 | 1 | 25 | 1 | 100 |
| post1 | 5 | 4 | 39 | 13 | 0 | - | 4 | 100 |
| post2 | 0 | 17 | 29 | 10 | 8 | 33 | 3 | - |

‘correctly named’ = objects correctly named twice or thrice during three naming sessions;

‘incorrectly named’ = objects named correctly once or never during three naming sessions.

**Table 3** Additional neuropsychological information for the eight aphasia patients

| **Test/Patient ID** | **P01** | **P02** | **P03** | **P04** | **P05** | **P06** | **P07** | **P08** |
| --- | --- | --- | --- | --- | --- | --- | --- | --- |
| **Mini Mental Status Test (max. score of 30)** |  |  |  |  |  |  |  |  |
| total | 23 | 25 | 18 | 24 | 21 | 17 | 25 | 17 |
| Errors presumably due to language impairment | 6 | 3 | 10 | 6 | 9 | 9 | 5 | 13 |
| **Digit span** |  |  |  |  |  |  |  |  |
| Forward (raw score/ span scores) | 0/ 2 | 2/ 4 | 0/# | 0/# | 0/2 | 1/3 | 2/3 | 5/5 |
| Backward(raw score/ span scores) | 0/ 1 | 1/ 2 | 0/# | 0/# | 0/2 | 2/2 | 1/2 | 3/3 |
| **Corsi blocks (raw score)** |  |  |  |  |  |  |  |  |
| forward | 5 | 7 | 7 | 6 | 10 | 4 | 9 | 7 |
| backward | 7 | 7 | 6 | 5 | 11 | 5 | 10 | 8 |
| **Rey figure (raw scores, max. 36)** |  |  |  |  |  |  |  |  |
| copy | 31 | 29 | 32 | # | 29 | 30 | 34 | 29 |
| recall after 30 min | 22 | 17 | 10 | # | 17 | 17 | 18 | 13 |
| **Benton test** |  |  |  |  |  |  |  |  |
| reproduction | 6 | 6 | 8 | # | 7 | 3 | 7 | 5 |
| number of errors | 10 | 6 | 4 | # | 3 | 10 | 5 | 8 |
| **Verbal paired associates** |  |  |  |  |  |  |  |  |
| number of trials | 6 | 6 | # | 5 | 6 | 6 | 6 | # |
| immediate recall (in 3 consecutive trials) | 4 | 5 | # | 10 | 8 | 4 | 15 | # |
| delayed recall | 2 | 3 | # | 6 | 5 | 0 | 6 | # |
| **Visual paired associates** |  |  |  |  |  |  |  |  |
| number of trials | 6 | 3 | 4 | 6 | 3 | 6 | 3 | 5 |
| immediate recall (in 3 consecutive trials) | 10 | 12 | 8 | 2 | 17 | 6 | 15 | 6 |
| delayed recall | 2 | 5 | 4 | 5 | 6 | 1 | 6 | 3 |
| **Word fluency test (items produced in 2 min)** |  |  |  |  |  |  |  |  |
| Letter [s] | 1 | 3 | 0 | 2 | 1 | 2 | 5 | # |
| Semantic category: professions | 3 | 9 | 3 | 6 | 5 | 0 | 6 | # |
| **HAWIE (Wechsler scores)** |  |  |  |  |  |  |  |  |
| Picture completion | 5 | 10 | 6 | 4 | 8 | 1 | 8 | 9 |
| Block design | # | 6 | 12 | # | 9 | # | 13 | 10 |
| **TMT - A (s/ PR)** | 110/ * | 40/ 10 | 80/ * | 87/ * | 45/ 10 | 150/ * | 35/ 20 | 71/ * |
| **TMT - B (s/ PR)** | # | # | 400/ * | # | 150/ * | # | 150/ * | 259/ * |
| **WCST – Nelson version (raw scores)** |  |  |  |  |  |  |  |  |
| hits (max. 48) | 41 | 36 | 32 | 26 | 33 | 28 | 23 | 20 |
| random errors | 6 | 8 | 15 | 13 | 11 | 11 | 21 | 25 |
| perseveration errors | 1 | 2 | 1 | 9 | 4 | 9 | 4 | 3 |
| **VAMS (T-score)** |  |  |  |  |  |  |  |  |
| afraid | 56 | 44 | 42 | 62 | 45 | 43 | 51 | 46 |
| confused | 57 | 44 | 41 | 52 | 73 | 42 | 43 | 45 |
| sad | 44 | 43 | 40 | 52 | 67 | 40 | 49 | 63 |
| angry | 44 | 43 | 42 | 49 | 89 | 42 | 48 | 50 |
| energetic | 64 | 63 | 45 | 35 | 45 | 64 | 49 | 50 |
| tired | 40 | 36 | 36 | 40 | 41 | 36 | 36 | 57 |
| happy | 60 | 60 | 60 | 61 | 23 | 59 | 60 | 47 |
| tense | 47 | 41 | 40 | 46 | 80 | 40 | 53 | 57 |

TMT = Trailmaking Test, HAWIE = Hamburg-Wechsler-Intelligenztest für Erwachsene (German Wechsler Adult Intelligence Scale), WCST = Wisconsin Card Sorting Test (Nelson), VAMS = Visual Analogue Mood Scales, n = normal, q = questionable, d = deviant, # = not accomplishable, * = floor effect (no norms available).

**Table 4** Regional activity during overt object naming for the healthy control subjects

| **Session 1** | | | | | |
| --- | --- | --- | --- | --- | --- |
| *** MNI-coordinates, Z-score** | ***x*** | ***y*** | ***z*** | ***Z*** | ***BA*** |
| **Language related areas** | | | | | |
| Left superior  temporal /inferior frontal gyrus | -63 | -42 | 18 | 4.66 | 22/44 |
| Right inferior  frontal/superior temporal gyrus/Insula | 57 | 12 | 18 | 3.86 | 44/47/38 |
| Right middle temporal gyrus | 50 | -55 | 0 | 3.81 | 37 |
| Right inferior  parietal gyrus | 63 | -42 | 33 | 4.20 | 40/2 |
| **Attention related areas** | | | | | |
| Left anterior cingulate gyrus | -3 | 12 | 9 | 4.05 | 25 |
| Right superior frontal gyrus | 15 | 18 | 57 | 4.05 | 6/10 |
| Right precuneus/  superior parietal gyrus | 33 | -69 | 39 | 3.80 | 7/19 |
| Left superior  parietal gyrus | -27 | -60 | 39 | 3.31 | 7 |
| **Motor areas** | | | | | |
| Left primary  motor area | -48 | -18 | 45 | 3.37 | 4 |
| Right putamen/  globus pallidus/  thalamus | 21 | -3 | 15 | 3.30 | - |
| **Memory related areas** | | | | | |
| Right para-hippocampal  gyrus | 18 | -24 | -18 | 4.13 | 34/35 |
| **Visual areas** | | | | | |
| Left/Right occipital lobe | -15 | -82 | -1 | 4.07 | 18/19 |
| Right cuneus | 3 | -84 | 18 | 3.50 | 18 |

* Clusters exceeding a single-voxel *p* value of 0.001 for a minimum of 10 voxels are reported. *BA* = Brodmann area.

**Table 5** Regional activity during overt naming of trained objects for the chronic aphasia patients

| **Prior to training** | | | | | |
| --- | --- | --- | --- | --- | --- |
| *** MNI-coordinates, Z-score** | ***x*** | ***y*** | ***z*** | ***Z*** | ***BA*** |
| **Language and motor related areas** | | | | | |
| Right supplementary motor area/Insula | 48 | -6 | 36 | 3.51 | 6/13 |
| **Attention related areas** | | | | | |
| Right posterior cingulate gyrus | 3 | -45 | 3 | 3.92 | 29 |
| Right inferior frontal gyrus | 45 | 23 | -4 | 3.54 | 47 |
| **Memory related areas** | | | | | |
| Left  parahippo-campal gyrus | -9 | -30 | -12 | 3.05 | 30 |
| **Visual areas** | | | | | |
| Left/Right occipital lobe | -3 | 6 | 57 | 5.14 | 19 |

| **Immediately after training** | | | | | |
| --- | --- | --- | --- | --- | --- |
| *** MNI-coordinates, Z-score** | ***x*** | ***y*** | ***z*** | ***Z*** | ***BA*** |
| **Language and motor related areas** | | | | | |
| Right primary motor cortex | 21 | -27 | 72 | 3.40 | 4 |
| Right middle/  superior temporal gyrus | 57 | -27 | -3 | 3.56 | 21/22 |
| **Attention related areas** | | | | | |
| Right medial frontal/  cingulate gyrus | 6 | -3 | 63 | 3.84 | 6/32 |
| Right posterior cingulate cortex | 0 | -57 | 0 | 3.34 | 30 |
| **Visual areas** | | | | | |
| Left occipital lobe | -15 | -69 | -21 | 4.20 | 18/19 |
| Right occipital lobe | 42 | -60 | -24 | 3.50 | 37/20 |

| **Eight months after training** | | | | | |
| --- | --- | --- | --- | --- | --- |
| *** MNI-coordinates, Z-score** | ***x*** | ***y*** | ***z*** | ***Z*** | ***BA*** |
| **Language and motor related areas** | | | | | |
| Right superior temporal/  inferior frontal gyrus | 9 | -69 | -15 | 3.05 | 22/47 |
| Right insula/  supplementary motor area | 45 | -45 | 30 | 3.02 | 13/6 |
| Right posterior cingulate gyrus | 18 | 42 | 27 | 3.78 | 29 |
| **Visual areas** | | | | | |
| Right/Left occipital lobe | 3 | 3 | 18 | 3.97 | 19 |

* Clusters exceeding a single-voxel *p* value of 0.01 for a minimum of 10 voxels are reported. *BA* = Brodmann area.
