# Supplementary material for: Imaging short- and long-term training success in chronic aphasia
Source: BMC Neurosci. 2009 Sep 22;10:118. doi: 10.1186/1471-2202-10-118 (PMC2754483; doi:10.1186/1471-2202-10-118)
Supplement: Additional file 3 — Brain activity changes immediately after training and at the follow-up assessment. Brain activity changes in the patients and healthy controls: Brain areas showing a) positive and b) negative correlations between short-term training success and 'post1-pre' training activity changes for trained object names (masked with the respective results for untrained object names). Brain areas showing c) positive and d) negative correlations between long-term training success and 'post2-pre' training activity changes for trained object names (masked with the respective results for untrained object names). The corresponding results for the control group (Session1 versus Sesssion2) are displayed in italic font [file 1471-2202-10-118-S3.DOC]

**Additional file 3**

|  | **a)** | | | | | **b)** | | | | | **c)** | | | | | **d)** | | | | |
| --- | --- | --- | --- | --- | --- | --- | --- | --- | --- | --- | --- | --- | --- | --- | --- | --- | --- | --- | --- | --- |
| ***MNI-coordinates, Z-score** | ***x*** | ***y*** | ***z*** | ***Z*** | ***BA*** | ***x*** | ***y*** | ***z*** | ***Z*** | ***BA*** | ***x*** | ***y*** | ***z*** | ***Z*** | ***BA*** | ***x*** | ***y*** | ***z*** | ***Z*** | ***BA*** |
| **Memory related areas** | | | | | | | | | | | | | | | | | | | | |
| Left para-hippocampal gyrus/  hippocampus | -15  -30 | -42  -9 | -18  -18 | 3.76  3.12 | 36 |  |  |  |  |  |  |  |  |  |  |  |  |  |  |  |
| Right para-  hippocampus | 24 | -24 | -12 | 3.45 | 28 |  |  |  |  |  |  |  |  |  |  |  |  |  |  |  |
| **Attention related areas** | | | | | | | | | | | | | | | | | | | | |
| Right precuneus/  Cingulate gyrus | 21 | -51 | 51 | 3.69 | 7/31 |  |  |  |  |  |  |  |  |  |  |  |  |  |  |  |
| **Language related areas** | | | | | | | | | | | | | | | | | | | | |
| Right middle temporal gyrus |  |  |  |  |  |  |  |  |  |  | 57 | -30 | -3 | 3.88 | 21/22 |  |  |  |  |  |
| Right inferior parietal cortex |  |  |  |  |  | *60* | *-42* | *39* | *3.69* | *40* |  |  |  |  |  | 39 | -36 | 39 | 3.79 | 40 |
| **Motor areas** | | | | | | | | | | | | | | | | | | | | |
| Left suppl. motor area |  |  |  |  |  |  |  |  |  |  |  |  |  |  |  | -48 | -6 | 18 | 4.03 | 6 |
| Right caudate nucleus |  |  |  |  |  |  |  |  |  |  |  |  |  |  |  | 12 | -3 | 21 | 3.30 |  |
| **Visual areas** | | | | | | | | | | | | | | | | | | | | |
| Right occipital lobe (fusiform gyrus) | 33 | -69 | -12 | 3.72 | 19 |  |  |  |  |  |  |  |  |  |  | 24 | -72 | -27 | 3.30 | 19 |
| Left occipital lobe (fusiform gyrus) | -30 | -75 | -6 | 3.23 | 19/37 |  |  |  |  |  |  |  |  |  |  |  |  |  |  |  |

* Threshold: single-voxel *p* value of < 0.05 for a minimum of 10 voxels within significant clusters (p< 0.05), including at least one voxel with an uncorrected single-voxel *p* value of $\leq$ 0.001 (*Z-*score $\geq$3.00) are reported. BA = Brodmann area. Results of the control group (displayed in italic font) are thresholded at a single voxel level of p< 0.005 (only voxels within significant clusters are reported, p< 0.05)
